# Viral rescue of magnocellular vasopressin cells in adolescent Brattleboro rats ameliorates diabetes insipidus, but not the hypoaroused phenotype

**DOI:** 10.1038/s41598-019-44776-1

**Published:** 2019-06-03

**Authors:** K. C. Schatz, L. M. Brown, A. R. Barrett, L. C. Roth, V. Grinevich, M. J. Paul

**Affiliations:** 1 Department of Psychology, University at Buffalo, SUNY, Buffalo, 14260 NY USA; 2 Department of Molecular Neurobiology, Max Planck Institute for Medical Research, Heidelberg, 69120 Germany; 3 Schaller Group on Neuropeptides, German Cancer Research Center, Heidelberg, 69120 Germany; 4 Department of Neuropeptide Research for Psychiatry, Central Institute of Mental Health, Heidelberg University, Mannheim, 68159 Germany; 5 Neuroscience Program, University at Buffalo, SUNY, Buffalo, 14260 NY USA; 6 Evolution, Ecology and Behavior Program, University at Buffalo, SUNY, Buffalo, 14260 NY USA; 7 Present Address: Letten Centre and GliaLab, Department of Physiology, Institute of Basic Medical Sciences, University of Oslo, 0317 Oslo, Norway

**Keywords:** Neuroscience, Neural circuits

## Abstract

Dysregulated arousal often accompanies neurodevelopmental disorders such as attention deficit hyperactivity disorder and autism spectrum disorder. Recently, we have found that adolescent homozygous Brattleboro (Hom) rats, which contain a mutation in the arginine vasopressin (AVP) gene, exhibit lower behavioral arousal than their heterozygous (Het) littermates in the open field test. This hypoaroused phenotype could be due to loss of AVP in magnocellular cells that supply AVP to the peripheral circulation and project to limbic structures or parvocellular cells that regulate the stress axis and other central targets. Alternatively, hypoarousal could be a side effect of diabetes insipidus – polydipsia and polyuria seen in Hom rats due to loss of AVP facilitation of water reabsorption in the kidney. We developed a viral-rescue approach to “cure” magnocellular AVP cells of their Brattleboro mutation. Infusion of a recombinant adeno-associated virus (rAAV) containing a functional *Avp* gene and promoter (rAAV-AVP) rescued AVP within magnocellular cells and fiber projections of the paraventricular nucleus of the hypothalamus (PVN) of male and female adolescent Hom rats. Furthermore, water intake was markedly reduced, ameliorating the symptoms of diabetes insipidus. In contrast, open field activity was unaffected. These findings indicate that the hyporaoused phenotype of adolescent Hom rats is not due to the loss of AVP function in magnocellular cells or a side effect of diabetes insipidus, but favors the hypothesis that central, parvocellular AVP mechanisms underlie the regulation of arousal during adolescence.

## Introduction

Several neurodevelopmental disorders are associated with altered arousal. Attention deficit hyperactivity disorder (ADHD) is characterized by hyperactivity and dysregulated physiological arousal. The latter typically presents as decreased autonomic and cortical arousal^1–3^. In addition, the social deficits of autism spectrum disorder (ASD) are often accompanied by increased stress and autonomic reactivity during social interactions^4,5^. Notably, increased skin conductance level (a measure of autonomic activity) is evident in 2-year old toddlers with ASD and is positively correlated with restrictive and repetitive behaviors^6^. The extent and direction of dysregulated arousal in ADHD and ASD can vary depending on age, gender, or disorder subtype^7–9^. Hence, determining the mechanisms that regulate arousal during development is important for our understanding of both normative and pathological development.

Arginine vasopressin (AVP) plays an important role in the development of several social and affective behaviors (reviewed in^10,11^), and some evidence suggests there may be a link between altered AVP function and neurodevelopmental disorders, including ASD, ADHD, and schizophrenia. For example, gene polymorphisms in the promoter region of AVP receptors have been associated with ASD (AVPR1a gene^12–14^) and ADHD (AVPR1b gene^15^). AVP also regulates fluid balance through its action on the V2 receptor in renal collecting tubule cells of the kidney to facilitate fluid retention^16,17^. Altered peripheral AVP and elevated levels of polydipsia have been noted in schizophrenics^18–21^. Notably, individuals with familial central diabetes insipidus, polydipsia and polyuria due to a mutation in the AVP gene, exhibit deficits in sustained attention^22,23^. In addition, one study found increased prevalence of ADHD in a small cohort of children with congenital nephrogenic diabetes insipidus, polydipsia and polyuria due to decreased sensitivity of the kidneys to AVP^24^.

Brattleboro rats can be used as a model to study the effects of life-long AVP disruption. Brattleboro rats have a single base pair deletion in the second exon of the *Avp* gene^25^. Similar to humans with AVP gene mutations, rats homozygous for the Brattleboro mutation (Hom) exhibit central diabetes insipidus characterized by striking polydipsia and polyuria^26^. Hom rats also exhibit atypical social behavior and altered behavioral arousal during development. Adolescent Hom rats display a hypoaroused/low anxiety-like phenotype compared to their Het littermates characterized by decreased locomotor activity in the open field test and decreased marble burying^27^. This hypoaroused phenotype may contribute to their atypical social behavior – decreased active social behaviors (e.g. social play and 50 kHz USVs) and increased passive social behaviors (e.g. huddling) during a social interaction test^27,28^.

The hypoaroused phenotype of Hom rats could be due to disruptions in central and/or peripheral actions of AVP. AVP acts through distinct pathways in the brain to regulate stress, autonomic function, anxiety, and circadian rhythmicity^29^, and disruptions to each of these pathways could impact arousal. Nevertheless, AVP receptors are located throughout the periphery^30^, including in tissues that could provide feedback to the brain to influence social behaviors and behavioral state^31^. AVP acts on V1a receptors located in blood vessels and in the area postrema, a circumventricular organ, to influence blood pressure^32,33^. The absence of AVP action on these receptors in Hom rats is thought to activate compensatory sympathetic mechanisms to normalize blood pressure of Hom rats^34^. In addition, the diabetes insipidus of Hom rats could be accompanied by side effects that impact arousal. For example, the loss of AVP action on V2 receptors in the kidneys could influence behavioral state due to a chronic partial dehydrated state. Consistent with peripheral actions of AVP on arousal, intraperitoneal administration of AVP induces rats to lay side-by-side, a passive social behavior characterized by inactivity^35^.

In wild type rats, endogenous peripheral AVP is supplied by magnocellular cells of the paraventricular (PVN) and supraoptic (SON) nuclei of the hypothalamus, which project to the posterior pituitary where AVP is released into the peripheral circulation^36^. AVP magnocellular cells of the PVN also project to the limbic areas including the lateral habenula, amygdala, and hippocampus^37–41^, which could also impact affective behaviors or arousal. The PVN also contains two types of parvocellular AVP cells: one projects to the external zone of the median eminence to regulate the stress response and the other projects to hindbrain nuclei to regulate the autonomic nervous system^42–44^. In the present study, we used the Brattleboro rat model to test the hypothesis that magnocellular AVP cells regulate behavioral arousal. Brattleboro rats have functional AVP receptors^45,46^ and retain sensitivity to AVP and AVP agonists^47^. Therefore, we designed a recombinant adeno-associated virus carrying a functional *Avp* gene driven by an AVP promoter (rAAV-AVP) to selectively rescue AVP production in PVN magnocellular AVP cells of adolescent Hom rats. We then tested the impact of rescuing AVP function in this pathway on water intake and behavioral arousal during adolescence. Here, we show that 1) a conserved AVP promoter, 1.9 kb in length, restricts expression of viral constructs to magnocellular AVP cells of the PVN and 2) infusion of an rAAV equipped with the open reading frame sequence of the *Avp* gene under the control of this AVP promoter rescues AVP production in magnocellular PVN cells of Hom rats, including AVP transport through projection fibers to limbic areas. We further show that 3) this viral rescue of PVN magnocellular AVP ameliorates the diabetes insipidus of Hom rats, but 4) does not reverse the decrease in behavioral arousal of Hom rats.

## Results

### Viral rescue of AVP production and function to PVN magnocellular AVP cells of adolescent Brattleboro rats

**Figure 1:**
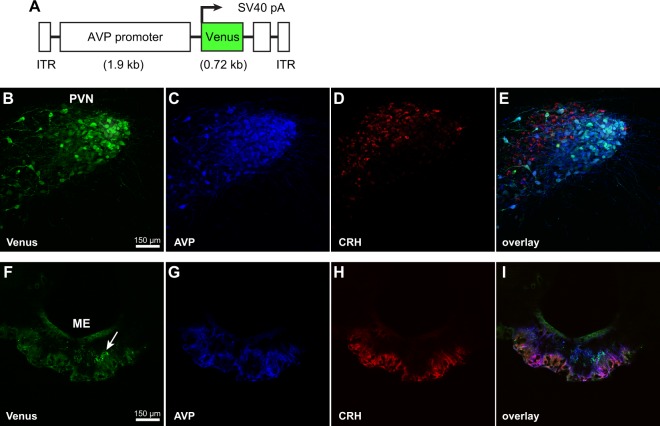
The 1.9 kb AVP promoter drives Venus fluorescence in magnocellular AVP cells of the PVN. (**A**) Schematic illustration of the recombinant adeno-associated virus (rAAV) containing a 1.9 kb AVP promoter driving a Venus marker (rAAV-Venus). (**B**–**I**) Confocal images of triple-immunofluorescent staining for Venus (green), AVP (blue), and CRH (red) within the PVN and median eminence (ME) of an adult adrenalectomized Wistar rat that received an infusion of the rAAV-Venus into the PVN. CRH staining delineates the parvocellular compartment of the PVN and the external zone of the median eminence. Light blue staining signifies co-localization of AVP and Venus; magenta staining signifies co-localization of AVP and CRH. Note that Venus expression is largely restricted to the magnocellular compartment of the PVN and the internal zone of the median eminence, the latter indicated by the white arrow. Note the absence of co-localization of Venus and CRH immunosignals within cells of the parvocellular region of the PVN and the external zone of the median eminence.

We first generated an rAAV equipped with the fluorescent marker, Venus, under the control of a conserved AVP promoter that spans 1.9 kb upstream of the transcription start site (ATG) of the *Avp* gene (rAAV-Venus; Fig. 1A). This rAAV has previously been shown to drive the Venus fluorescent marker in AVP cells of the PVN in Wistar rats^48^. Using CRH as a marker for parvocellular PVN cells and the rAAV-Venus^49–53^, we asked whether the 1.9 kb AVP promoter drives expression of Venus in parvocellular and/or magnocellular AVP cells of the PVN. Six adult male Wistar rats received infusions of the rAAV-Venus in the PVN. One week after the infusion of the rAAV-Venus, half of the rats were adrenalectomized to increase CRH production, thereby enhancing visualization of CRH PVN neurons after immunohistochemstry. Three weeks after the infusion, rats were sacrificed and brain tissue was processed for triple-label immunofluorescence for Venus, AVP, and CRH.

Venus was detected in AVP cells and fibers of the PVN and median eminence of adrenalectomized (Fig. 1B–I) and intact (not illustrated) rats. In both adrenalectomized and intact rats, Venus staining was largely restricted to the magnocellular compartment of the PVN and the internal zone of the median eminence. There were a few Venus-immunoreactive (ir) cell bodies detected in the parvocellular compartment of the PVN. Nevertheless, we failed to detect any co-localization of Venus-ir and CRH-ir cells in adrenalectomized or intact rats. We cannot rule out the possibility that a subpopulation of parvocellular cells do not produce detectable CRH, even after adrenalectomy. Nevertheless, the present data indicate that the 1.9 kb AVP promoter predominantly drives expression in magnocellular cells of the PVN.

**Figure 2:**
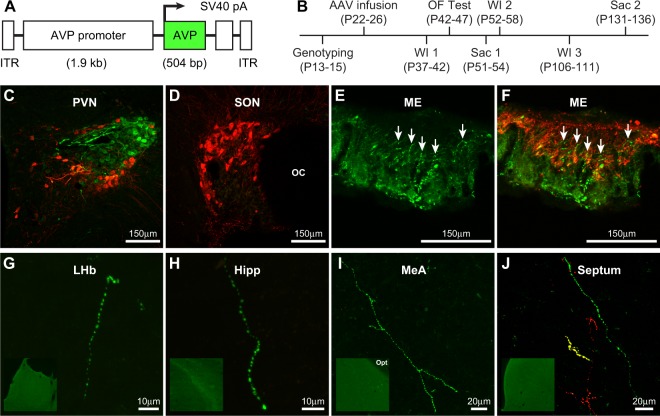
Viral rescue of AVP production in PVN cells and fiber projections of Brattleboro rats. (**A**) Schematic illustration of the rAAV containing a conserved AVP promoter driving expression of a functional *Avp* gene (rAAV-AVP). (**B**) Timeline of experimental procedures. OF = open field; WI 1, WI 2, and WI 3 = 3 time points of water intake measures; Sac 1 and Sac 2 = times of sacrifice, note that the subset of animals used to test the longevity of the virus were sacrificed at Sac 2. (**C–F**) Confocal images of double-immunofluorescent staining for AVP (green) and oxytocin (red) within the PVN, SON, and median eminence (ME) of a Hom-AVP rat. Oxytocin staining was used to identify the PVN, SON, and internal zone of the ME of Hom rats. Note that AVP staining is present in the PVN, the target of the rAAV-AVP infusion, but not the SON. Arrows in panels E and F highlight AVP fibers in the internal zone of the ME. (**G–J**) Immunfluorescent staining of AVP fibers in the (**G**) lateral habenula (LHb), (**H**) hippocampus (Hipp), (**I**) medial amygdala (MeA), and (**J**) septum. Panel J shows a rare occurrence in which AVP and oxytocin were co-localized in the same fiber (yellow). Opt = optic tract. OC = optic chiasm.

We next constructed an rAAV containing the 1.9 kb conserved AVP promoter and the wild type, functional *Avp* gene (rAAV-AVP; Fig. 2A) and asked whether this rAAV-AVP could restore AVP production and function to PVN AVP cells of adolescent Hom Brattleboro rats. Male and female Hom rats received bilateral infusions of the rAAV-AVP directed at the PVN (Hom-AVP rats) between postnatal day (P)22-26, and 24-h water intake (WI) was measured between P37-42. Twenty-four-hour WI of Hom-AVP rats was compared to adolescent Hom and Het rats receiving the rAAV-Venus as a control virus (Hom-Venus and Het-Venus rats, respectively). Rats were sacrificed at either P51-54 or P131-136, and brains of the Hom-AVP rats were processed for AVP and oxytocin double-immunofluorescence to assess restoration of AVP peptide production in the PVN; oxytocin staining was used to identify the PVN and internal zone of the median eminence of Hom rats. Note that a subset of rats was sacrificed at P131-136 in order to test the longevity of the virus’s impact on water intake (see below). A timeline of experimental procedures is illustrated in Fig. 2B.

AVP immunosignal was detected within PVN, but not SON, cell bodies and fibers of Hom-AVP rats (Fig. 2C,D). AVP-ir fibers projected laterally across the anterior hypothalamus, and then arced downward, eventually reaching the internal zone of the median eminence (Fig. 2E,F). Using oxytocin to delineate the internal and external zones of the median eminence, we found that rescue of AVP fibers was largely restricted to the internal zone. Occasional AVP and oxytocin fiber-like staining was noted in the external zone. This pattern of fiber staining indicates that rAAV-AVP injections rescued AVP production and transport along the neurohypophysial tract that leads to the posterior pituitary. Infusion of the rAAV-AVP also rescued AVP within extra-neurohypophysial projections of Hom-AVP rats. Sparse AVP-ir fibers were detected within the lateral habenula, hippocampus, amygdala, and septum (Fig. 2G–J). These extra-neurohypophysial AVP-ir fibers exhibited the beaded, “string-of-pearls” appearance considered to be axon-like projections^54^. In all but a few rare cases, AVP-ir fibers were not immunoreactive for oxytocin (but see Fig. 2J).

**Figure 3:**
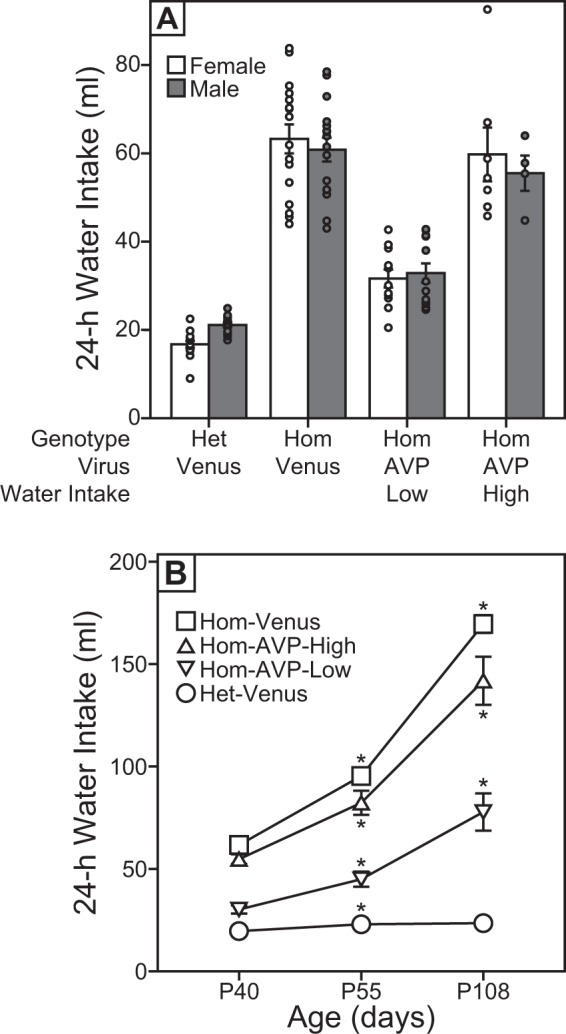
Viral rescue of peripheral AVP function of magnocellular PVN cells in adolescent Brattleboro rats. (**A**) Distribution (circles) and mean ± s.e. of 24-h water intake (WI) of Het-Venus, Hom-Venus, Hom-AVP-low drinkers and Hom-AVP-high drinkers recorded on P40. (**B**) Mean ± s.e. 24-h WI recorded across development in a subset of male and female Het-Venus, Hom-Venus, Hom-AVP-low drinker, and Hom-AVP high drinker rats. Asterisks indicate significant increase over previous time point (p ≤ 0.004, Bonferroni correction).

rAAV-AVP infusions ameliorated the diabetes insipidus of male and female adolescent Hom rats. Hom-AVP rats drank significantly less water than Hom-Venus rats (mean ± s.e. = 40.7 ± 2.65 ml vs. 62.0 ± 2.11 ml, p < 0.001, Tukey-Kramer Test), but more than Het-Venus rats (mean ± s.e. = 40.7 ± 2.65 ml vs. 18.8 ± 0.63 ml, p < 0.001, Tukey-Kramer Test); the latter indicates that restoration of peripheral AVP function was not complete. There was considerable variability in the 24-h WI of Hom-AVP rats (range = 20.5–92.6 ml). Using 24-h WI as a proxy measure for peripheral AVP function, we divided animals into low and high drinkers in order to identify individuals for which the rAAV-AVP infusion was most effective in restoring peripheral AVP function (Fig. 3A; Hom-AVP-low drinkers = 12 males and 11 females; Hom-AVP-high drinkers = 4 males and 7 females). Low drinkers were defined as Hom-AVP rats with WI measures below the range of same-sex Hom-Venus rats, and high drinkers were defined as rats with WI measures within the range of same-sex Hom-Venus rats. The low drinking group exhibited a 69% recovery of peripheral AVP function, whereas the high drinking group exhibited a 13% recovery (% recovery = [(24-h WI of Hom-Venus rats - 24-h WI of low or high drinking Hom-AVP rats)/(24-h WI of Hom-Venus rats - 24-h WI of Het-Venus rats)] * 100).

The longevity of decreased WI of Hom-AVP rats was assessed in a subset of rats, for which two additional WI measures were recorded at P52-58 and at P106-111 (Fig. 3B; 20 Het-Venus rats, 22 Hom-Venus rats, 11 Hom-AVP low drinking rats, 13 Hom-AVP high drinking rats). There was a main effect of genotype/virus condition and time on WI (p < 0.001, repeated-measures ANOVA) as well as an interaction between time and genotype/virus condition (p < 0.001, repeated-measures ANOVA). All groups increased their water intake between P42 and P55 (p < 0.001, Bonferroni correction), but only the Hom groups (Hom-Venus, Hom-AVP-low drinkers, and Hom-AVP-high drinkers) significantly increased water intake between P55 and P108 (Hom groups: p < 0.001; Het group: p > 0.999; Bonferonni correction). Hence, while Hom-AVP-low drinkers and Hom-AVP-high drinkers continued to drink less than their Hom-Venus littermates throughout the experiment (Hom-AVP-low drinkers or Hom-AVP-high drinkers vs. Hom-Venus, p ≤ 0.036, Bonferroni correction), the ability of the rAAV-AVP to restrain drinking appeared to decrease as these rats reached adulthood. Percent recovery of Hom-AVP low drinkers also decreased, albeit moderately, in this subset with age (% recovery of Hom-AVP low drinkers = 75%, 70%, and 63% at P40, P55, and P108, respectively). In the overall repeated-measures ANOVA, there was also a significant interaction between time and sex (p = 0.022, repeated-measures ANOVA), but post hoc tests were not significant (males vs. females at P40, P55, and P108, p ≥ 0.484 for all comparisons, Bonferroni correction).

### Viral rescue of peripheral AVP function does not impact behavioral arousal of adolescent Brattleboro rats

**Figure 4:**
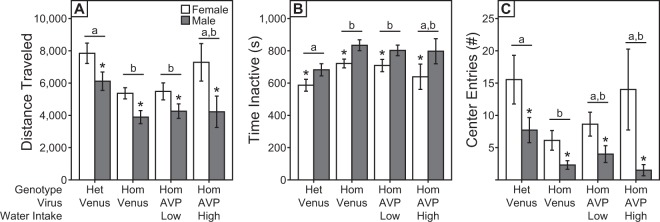
Viral rescue of peripheral AVP function does not impact behavioral arousal of adolescent Brattleboro rats. Mean ± s.e. (**A**) distance traveled, (**B**) time inactive, and (**C**) center entries of adolescent Het-Venus, Hom-Venus, Hom-AVP-low drinker, and Hom-AVP-high drinker rats tested in the open field test between P42-47. Groups with differing letters differ significantly from each other (p < 0.05, Tukey-Kramer test). Asterisks indicate significant main effect of sex (p < 0.001, two-way ANOVA).

We next asked whether this partial rescue of peripheral AVP function would impact the hypoaroused phenotype of adolescent Hom rats by assessing the open field activity on P42-47, during late-adolescence^55^. Consistent with our previous findings^27^, adolescent Hom-Venus rats traveled less distance in the open field (Het-Venus vs. Hom-Venus, p < 0.001, Tukey-Kramer Test), spent more time inactive (Het-Venus vs Hom-Venus, p < 0.001, Tukey-Kramer Test), and entered the center of the arena fewer times (Het-Venus vs Hom-Venus, p = 0.001, Tukey-Kramer Test) than their Het-Venus littermates (Fig. 4). In addition, females traveled a greater distance (main effect of sex, p < 0.001, two-way ANOVA), spent less time inactive (main effect of sex, p < 0.001, two-way ANOVA), and entered the center more times (main effect of sex, p < 0.001, two-way ANOVA) than males. Open field activity of adolescent Hom-AVP-low drinkers did not differ from Hom-Venus rats in any measure (distance traveled: p = 0.98, time inactive: p = 0.95, center entries: p = 0.60, Tukey-Kramer Test). Furthermore, Hom-AVP-low drinkers traveled less distance in the open field (Het-Venus vs Hom-AVP-low drinkers, p = 0.001, Tukey-Kramer Test) and spent more time inactive (Het-Venus vs Hom-AVP-low drinkers, p = 0.009, Tukey-Kramer Test) than Het-Venus rats. Hom-AVP low drinkers also tended to enter the center fewer times than Het-Venus rats, although the center entries measure was not significant (Het-Venus vs. Hom-AVP-low drinkers, p = 0.143, Tukey-Kramer Test). Similarly, Hom-AVP-high drinkers did not differ from Hom-Venus rats or Hom-low drinkers in any measure of open field activity (distance traveled: p > 0.11, time inactive: p > 0.33, center entries: p > 0.46, Tukey-Kramer Test). Activity measures of Hom-AVP-high drinkers also did not differ significantly from those of Het-Venus rats, largely due to intermediate values in females, higher variability, and low sample size in males.

**Figure 5:**
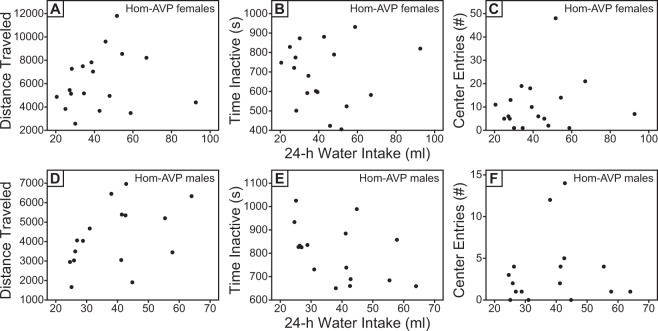
Behavioral arousal of adolescent Hom-AVP rats is not related to their peripheral AVP function. Correlational analyses for 24-h water intake with (**A**,**D**) distance traveled, (**B**,**E**) time spent inactive, and (**C**,**F**) number of center entries of male and female Hom-AVP rats. There were no significant correlations between 24-h water intake and any measure of open field activity (see text for details).

While these data suggest that peripheral AVP does not influence the hypoaroused phenotype of Hom rats, it remains possible that greater rescue of peripheral AVP is required to detect significant differences between mean activity values of Hom-AVP-low drinker and Hom-Venus groups. To further test the hypothesis that peripheral AVP modulates behavioral arousal, we took advantage of the variability in rescue of peripheral AVP function (i.e., 24-h WI) of Hom-AVP rats. If peripheral AVP influences arousal, then Hom-AVP rats with the greatest rescue of peripheral AVP function (i.e., lowest WI) should exhibit the highest levels of activity. Hence, we tested whether there was a correlation between WI and open field activity measures of Hom-AVP rats (including both low and high drinkers). Correlations were conducted separately for males and females due to the main effect of sex on all open field activity measures. Counter to the above prediction, 24-h WI did not correlate with distance traveled (Fig. 5A,D; females: p = 0.542, R^2^ = 0.024, linear regression; males: p = 0.088, R^2^ = 0.194, linear regression), time spent inactive (Fig. 5B,E; females: p = 0.966, R^2^ < 0.001, linear regression; males: p = 0.088, R^2^ = 0.194, linear regression), or center entries (Fig. 5C,F; females: p = 0.530, R^2^ = 0.025, linear regression; males: p = 0.756, R^2^ = 0.007, linear regression).

## Discussion

In the present study, we infused an rAAV containing a functional *Avp* gene construct driven by an AVP-specific promoter into the PVN of adolescent Hom Brattleboro rats to selectively “cure” AVP cells within this nucleus. Previous studies have used a similar approach to restore AVP in the SON of adult Brattleboro rats using a generalized, constitutive promoter (human cytomegalovirus promoter) that does not restrict gene expression to a particular cell phenotype^56–58^. Constructs containing at least 288 bp of the AVP promoter upstream of the transcription start site maintain cell-type specificity within AVP cells^59^. Consistent with this, the 1.9 kb promoter used in the present study restricts peptide production to AVP cells in the PVN of Wistar rats^48^. Here, we further show that this promoter selectively drives Venus fluorescence in magnocellular cells, with no detectable staining in parvocellular cells. When coupled with a functional AVP gene construct, this rAAV-AVP was able to rescue AVP production within PVN cell bodies and fiber projections of Hom Brattleboro rats. Furthermore, intra-PVN infusion of the rAAV-AVP ameliorated the polydipsia of Hom rats, markedly reducing, but not completely normalizing, WI. Hence, using this approach we were able to rescue AVP production and function to PVN magnocellular AVP cells, thereby rescuing peripheral AVP action, albeit partially.

We further asked whether intra-PVN infusions of the rAAV-AVP would impact behavioral arousal of adolescent Hom rats. Consistent with our previous findings^27^, adolescent Hom rats exhibited decreased behavioral arousal compared to their Het siblings, as measured by the number of center zone entries, distance traveled, and the amount of time spent inactive in the open field. In contrast to drinking behavior, however, infusion of the rAAV-AVP did not affect any measure of activity in the open field test. Using 24-h WI as a proxy measure for peripheral AVP action, we identified Hom-AVP rats with the greatest level of peripheral AVP rescue: Hom-AVP-low drinkers, which exhibited a 69% recovery of drinking behavior. Nevertheless, Hom-AVP-low drinkers did not differ from Hom-Venus rats in their open field activity, and continued to exhibit lower open-field activity than Het-Venus rats. Furthermore, WI of Hom-AVP rats did not correlate with any measure of open field activity, indicating that there was no relationship between the extent of peripheral AVP restoration and behavioral arousal. These data suggest that the hypoaroused phenotype of Hom rats is not due to the loss of peripheral AVP action and is not a side effect from their diabetes insipidus; e.g., general malaise or altered behavioral state due to disruptions in fluid balance. This is consistent with previous findings demonstrating that decreased daily running wheel activity of adult Brattleboro rats persists after restoration of peripheral AVP using osmotic minipumps^60^. Although the lack of correlation suggests there is no dose-response effect between peripheral AVP function and behavioral arousal, it remains possible that more complete restoration or restoration in both the PVN and SON is needed to impact open field activity. Current data, however, argue against a role for peripheral AVP in behavioral arousal and instead point to a predominantly central mechanism.

The hypoaroused phenotype of Hom rats is accompanied by a low anxiety-like phenotype. In addition to decreased open field activity, adolescent Hom rats exhibit decreased marble burying behavior compared to their Het littermates^27^. Similarly, adult Hom rats exhibit decreased anxiety-like behavior in the marble burying test and elevated plus maze^61,62^. AVP acts through central mechanisms to regulate anxiety (see^63^ and references therein). Blocking AVP action in the septum using a V1aR antagonist or V1R antisense oligonucleotides increases percentage of entries and time spent in the open arms of the elevated plus maze, an anxiolytic effect^64,65^. Rats selectively bred for high anxiety-like behavior (HAB rats) on the elevated plus maze exhibit increased AVP release and mRNA expression in the PVN compared to rats bred for low anxiety-like behavior (LAB rats^66^). Furthermore, anxiety-like behavior of HAB rats can be reduced by infusions of a V1aR antagonist into the PVN^66^. Given that anxiety is characterized, in part, by increased arousal^67,68^, AVP actions on these two behavioral states may be linked. Specifically, AVP may impact anxiety by modulating arousal or vice versa. Similar to the present results on behavioral arousal, decreased anxiety-like behavior of adult Hom rats persists after restoration of peripheral AVP action, here by subcutaneous administration of desmopressin^61^, a V2 and V1b agonist^69,70^. These findings further support the hypothesis that the hypoaroused, low anxiety-like phenotype of adolescent Hom rats is due to the loss of central actions of AVP.

In addition to AVP-ir within the neurohypophysial tract, we also detected AVP-ir fibers in the lateral habenula, hippocampus, amygdala, and septum of Hom-AVP rats after viral vector-based introduction of the functional *Avp* gene. These findings provide further evidence for extra-neurohypophysial projections of magnocellular AVP cells. AVP magnocellular projections to the hippocampus have been proposed to travel along three distinct routes, one being through a fimbria/fornix pathway that traverses the septum^37^. Rood, *et al*.^71^ also noted several divisions of the septum where sparse to moderate AVP-ir persisted in mice after gonadectomy (which eliminates AVP-ir originating from parvocellular cells of the BNST and MeA) and SCN lesions. Neurobiotin cell filling and retrograde tract tracing experiments indicate that PVN magnocellular AVP cells also project to the lateral habenula and amygdala, where they synapse on GABA interneurons^39–41^. The functional roles of these extra-neurohypophysial projections are not understood. Optogenetic manipulations of PVN AVP projections to the CA2 region of the hippocampus provide strong support for a role in social memory^72^. AVP magnocellular projections to the amygdala and the lateral habenula have been proposed to modulate anxiety and stress-coping behaviors^40,41^, but experiments conducted to date cannot rule out contributions from parvocellular AVP pathways that innervate these areas. If extra-neurohypophysial magnocellular AVP projections modulate anxiety and stress, one might expect they would also modulate activity in the open field test. Counter to this prediction, however, viral rescue of AVP in PVN magnocellular AVP cells did not impact the open field activity of Hom-AVP rats, suggesting that these projections are not sufficient to alter the hypoaroused phenotype of Brattleboro rats. Caution is warranted here, however, as AVP fiber projections within these brain areas arise from multiple sources in wild type rats^37,71^. Hence, we were not able to determine the extent of viral rescue for these magnocellular extra-neurohypophysial projections and assess potential correlations with open field activity.

There are several parvocellular AVP pathways in the brain^29,73^, each of which could influence arousal. Parvocellular cells of the PVN could influence arousal through their projections to 1) hindbrain structures that regulate the autonomic nervous system^42^ or 2) the median eminence where release of AVP augments the stress response at the level of the pituitary^44^. While the latter is ultimately a peripheral mechanism, the rAAV-AVP manipulation in the present experiment cannot rule out potential contributions from this pathway. Hom rats exhibit a blunted adrenocorticotrophic response to multiple types of stressors and this blunted response persists in the forced swim test even after restoration of peripheral AVP action using desmopressin^74^. Parvocellular AVP cells in the BNST and MeA project to hindbrain structures classically known to regulate arousal – the locus coeruleus and dorsal raphe^75–80^. These cell groups also project to the lateral septum^77,80^, where AVP modulates anxiety-like behavior on the elevated plus maze^64,65^. Finally, parvocellular AVP cells in the suprachiasmatic nucleus could impact the phase or amplitude of circadian rhythms in arousal^81,82^. Hom rats exhibit circadian neural, endocrine, and behavioral rhythms, but their free-running periods can be longer and their amplitudes blunted^83,84^. These circadian disruptions could result in shifted or blunted rhythms in arousal.

The Brattleboro rat is an ideal model for the development of gene therapy tools because the AVP deficiency is due to a single gene mutation and the effectiveness of the gene therapy can be assessed non-invasively by tracking water intake, urine output, or plasma osmolality. Previous studies have demonstrated that viral delivery of AVP cDNA behind the ubiquitous CMV promoter to the SON ameliorates the polydipsia, polyuria, and decreased plasma osmolality of adult male Hom rats^56–58^. The present experiment extends these findings to the PVN, AVP-specific promoters, females, and adolescents. Bienemann *et al*.^57^ and Ideno *et al*.^58^ were able to completely normalize baseline (but not stimulated) WI of Hom rats, whereas WI of Hom-AVP rats remained above typical Het levels in the present experiment. In the present study, incomplete recovery of 24-h water intake in Hom-AVP rats could be due to insufficient restoration of AVP in the PVN or the fact that only the PVN was targeted; full recovery may require AVP restoration within the SON. The use of an AVP-specific promoter restricts gene expression to AVP cells and is presumably under more natural regulation than the strong constitutive and ubiquitous CMV promoter. In the future, expression levels of AVP can be enhanced via Cre-dependent amplification systems; e.g., a combinatorial approach using an rAAV driving Cre-recombinase under the AVP promoter and a Cre-dependent rAAV carrying a FLEX *Avp* gene.

Unlike studies in adults where the level of rescued drinking remained constant for the duration of the experiment (4 months to over a year depending on the experiments^56–58^), drinking increased in Hom-AVP rats in the present study as the rats became adults. This raises several potential challenges that are unique to gene therapy studies conducted during development. In some brain areas, cell death could lead to the loss of the AAV, and cell division might dilute AAV expression. In addition, global developmental physiological changes could alter the effectiveness of the virus. For example, in the present experiment, increased fluid balance demands of a growing body may have rendered the level of AVP expression during adolescence insufficient for the larger adult. The present findings highlight the need for studies testing the effectiveness of viral approaches in developmental paradigms. These studies are critical for the development of gene therapies for the treatment of diseases and disorders in children and adolescents.

Recently, there has been renewed interest in the role of AVP in postnatal development^10,11^. AVP impacts numerous social and affective behaviors in juveniles and adolescents – social play, social memory, prosocial communication, huddling, behavioral arousal, and anxiety-like behavior^27,85–88^. In addition, AVP has been implicated in developmental disorders, including those that impact arousal, social behaviors, and emotion regulation (e.g., ADHD and autism spectrum disorders^12,15^). The available evidence indicates that AVP’s role in development is complex. Pharmacological manipulations of AVP can have opposite actions depending on the sex of the animal or the brain region manipulated^86^. Furthermore, AVP’s effect can be dependent on the context in which the animal is tested^89^. This complexity likely arises from AVP’s distinct actions across numerous neural pathways; e.g., circadian, autonomic, stress, sensory, and social^29^. The Brattleboro rat provides a useful model for studying the impact of lifelong disruptions to AVP (e.g.^27,28^). The major limitation of this model, however, is that the global loss of AVP prevents the assessment of individual AVP pathways. The present study demonstrates a new approach that overcomes this limitation – selectively rescuing an individual AVP pathway. This approach asks whether a given pathway is sufficient to maintain a particular behavior and complements pharmacological and knockdown studies in wild type rats, which ask if a particular pathway is necessary. Here, we demonstrate that our rAAV-AVP rescues AVP production in magnocellular cells of the PVN. Furthermore, rescue of AVP production in this pathway ameliorated the polydipsia of adolescent Brattleboro rats. Nevertheless, this manipulation did not affect open field activity, suggesting that AVP acts predominantly through parvocellular pathways to regulate behavioral arousal during adolescence. Future design of promoter constructs or combinatorial viral approaches that drive AVP expression in parvocellular AVP cells of the PVN and other nuclei will provide valuable tools to manipulate other AVP pathways both in Brattleboro and wild type models. These studies will help uncover the role of individual AVP pathways in normative and pathological development.

## Methods

### Subjects

Six adult male Wistar rats purchased from Charles River (Sulzfeld, Germany) were used to determine whether the AVP promoter drives peptide production in parvocellular or magnocellular AVP PVN cells. For the remaining experiments, male and female rats heterozygous (Het; male n = 14, female n = 15) or homozygous (Hom; male n = 32, female n = 34) for the Brattleboro mutation were obtained from our local breeding colony, which was derived from rats originally obtained from the Rat Resource and Research Center (University of Missouri, Columbia, MO). Brattleboro subjects were generated from 15 Hom male x Het female breeding pairs in order to generate Het and Hom offspring in each litter. Rats were genotyped on postnatal day (P)13-15 and weaned into same-sex, same-genotype pairs on P21. Rats were housed in plastic cages (44 cm × 22.5 cm × 20.5 cm) with corn cob bedding (Envigo). Food and water were available *ad libitum*. The lights and ambient temperature were maintained on a 12 h light/12 h dark cycle (lights off at 5 pm EST) and at 23 °C, respectively. Experiments were conducted in accordance with the *Guide for the Care and Use of Laboratory Animals* and were approved by the Animal Care and Use Committee at the University of Heidelberg (adult Wistar rat experiment) or the University at Buffalo, State University of New York (all other experiments).

### Genotyping

Genotyping was performed using the procedure developed by Paul *et al*.^28^. Briefly, ear tissue was collected from rats on postnatal day (P)13-15. The tissue was digested and DNA extracted using the REDExtract-N-Amp Tissue PCR Kit (SigmaAldrich). The DNA surrounding the Brattleboro deletion was amplified by PCR using the forward primer GACGAGCTGGGCTGCTTC, and reverse primer, CCTCAGTCCCCCACTTAGCC. The PCR product was incubated at 37 °C for 24 h with the restriction endonuclease BCG1 (New England BioLabs), which cuts the mutant, but not the wild type allele. Samples were then run on a 2% agarose gel using gel electrophoresis to visualize bands representing the wild type and mutant alleles. This method has been validated by sequencing^28^ and behavioral phenotyping of water intake^90^.

### Viruses and Injection Surgeries

rAAVs (serotype 1/2) carrying a conserved 1.9 kb AVP promoter and cDNA of either a fluorescent Venus tag (rAAV-Venus; Fig. 1A) or the AVP coding region (using 504 bp DNA containing open reading frame from Avp [NM_016992] Rat Tagged ORF, Origene Cat# RR211162; rAAV-AVP; Fig. 2A) were cloned and produced as described previously^48^. The conserved AVP promoter comprises a 1.9 kb sequence revealed by BLAT. Rats were anesthetized with isoflurane vapors (Piramal Healthcare, PA, USA) and mounted on a stereotaxic apparatus with the head level. Bilateral PVN injections of either the rAAV-AVP or the rAAV-Venus (300 nl/injection of undiluted virus) were made using a Hamilton syringe (Hamilton, NV, USA). The injections were made 1.5 mm caudal to bregma, 0.4 mm lateral to the midline, and 7.2 mm ventral to the skull for juveniles and 1.8 mm caudal to bregma, 0.4 mm lateral to the midline, and 8.0 mm ventral to the skull for adults. The skin was closed with wound clips (Braintree Scientific), which were removed 10 days after surgery.

### Fluorescence Immunohistochemistry

Rats were perfused with physiological saline or 1x PBS followed by 4% paraformaldehyde. Brains were sectioned on a vibratome (AVP promoter expression experiment, 50 µm) or microtome (viral rescue experiments, 40 µm) and stained with combinations of antibodies against GFP (detects Venus; Abcam; ab 13970 chicken; 1:10.000), vasopressin-Neurophysin (1:300; mouse; provided by Harold Gainer^91^, AVP (1:30,000; rabbit, T-4563, Peninsula Labs), and CRH (1:2000; Guinea pig, T-5007, Peninsula Labs) antibodies. The signals were visualized with Alexa 488 (1:1200), FITC (1:500), CY3 (1:500 or 1:1200), or CY5 (1:500) conjugated antibodies (Jackson Immuno-Research Laboratories, Inc). All images were acquired on a Nikon Eclipse Ni-U, Zeiss Axio Imager M1 light, Zeiss 710 confocal laser-scanning microscope, or Leica TCS SP5 confocal laser-scanning microscope.

### Water intake measures

Water intake (WI) was measured to assess the level of restoration of peripheral AVP function. Rats were separated into individual housing conditions. Water bottles were weighed, carefully placed on the cages for 24 h, and then weighed again. The difference in weight was recorded as the measure of WI; 1 g decrease = 1 ml water. A single 24-h WI measure was recorded before the open field test (P37-42) to minimize potential effects of isolation on behavioral testing. For a subset of animals, WI measures were extended into late adolescence (~P55) and adulthood (~P108) to test the longevity of the effects of the rAAV-AVP. The P55 time point was an average of three successive 24-h WI measures which ended between P52-P58, and the P108 time point was an average of two successive measures which ended between P106-111.

### Open field test

Open field tests were conducted in an arena (73.7 cm × 72.4 cm) inside a light- and sound-proof chamber (98 cm × 77 cm × 108 cm). Rats were placed in the arena and allowed to explore freely under bright white light for 20 min. Behavior was recorded by a camera mounted above the arena. Total distance traveled, time spent inactive, and number of center zone entries were scored automatically using EthoVision software (Noldus Information Technology Inc., Wageningen, The Netherlands). The center zone was defined as 25% of the arena and the surround zone was the outer 75% of the arena.

### Statistical analyses

Data were analyzed using a two-way analysis of variance (ANOVA) with sex and genotype/virus condition (Hom-AVP, Hom-Venus, Het-Venus) as independent variables. Data analyzed by ANOVA were tested for normality and homogeneity of variance using the Shapiro-Wilk and Levene’s test, respectively. A square root transformation was applied to center entries data for the open field test to correct a violation of the assumption of normality. Statistical analyses were conducted on the transformed center entries data, but graphs were constructed using the untransformed data. Tukey-Kramer post hoc tests were conducted when significant main effects or interactions were detected. Repeated measures ANOVAs were conducted using the Huynh-Feldt correction when Mauchley’s test for sphericity was significant. Post hoc tests for repeated measures ANOVAs were conducted with Bonferroni corrections. Correlations were analyzed using linear regression. All analyses were conducted using SPSS v23.0 (IBM). Significance was assumed when p < 0.05.

## Data Availability

The datasets generated during and/or analyzed during the current study are available from the corresponding author (KCS) on reasonable request. Request for viral vectors should be sent to VG.
